# Factors underlying inadequate parents’ awareness regarding pediatrics immunization: findings of cross-sectional study in Mosul- Iraq

**DOI:** 10.1186/1471-2431-14-29

**Published:** 2014-01-31

**Authors:** Omer Qutaiba B Al-lela, Mohd Baidi Bahari, Muhannad RM Salih, Mustafa G Al-abbassi, Ramadan M Elkalmi, Shazia Q Jamshed

**Affiliations:** 1 School of Pharmacy, Faculty of Medical Sciences, University of Duhok (UOD), Duhok, Iraq; 2 Faculty of Pharmacy, AIMST University, Kedah, Malaysia; 3 Pharmacy Department, Al-Rashed University College, Baghdad, Iraq; 4 Pharmacy College, Al-Mustansaria University, Baghdad, Iraq; 5 International Islamic University Malaysia, Kulliyyah of Pharmacy, Pahang, Malaysia

**Keywords:** Immunization, Iraq, Parents, Knowledge, Practice

## Abstract

**Background:**

Since last 100 years, immunization rate is one of the best public health outcome and service indicators. However, the immunization system is still imperfect; there are many countries that still have unvaccinated children. Parental decisions regarding immunization are very important to improve immunization rate. The aim of this study is to evaluate the association between parental knowledge-practice (KP) regarding immunization with family and immunization providers’ factors.

**Methods:**

This is a prospective cross-sectional study design. Immunization knowledge and practices among 528 Iraqi parents were evaluated through validated questionnaire. Familial data and immunization provider’s characteristics were collected from parents through interview.

**Results:**

More than half of respondents/study population (66.1%) have adequate knowledge- practice scores. Significant associations were noted for knowledge-practice groups with father’s education level, mother’s education level, mother’s age at delivery, number of preschool children, parents gender, family income, provider types, and birth place (*p* < 0.05).

**Conclusion:**

Immunization campaigns and awareness are required to improve parents’ knowledge and practice regarding immunization. The study results reinforce recommendations for use of educational programmes to improve the immunization knowledge and practice.

## Background

Knowledge and practice about children’s vaccines, communication about the risks and benefits of vaccines, disease risks, and other vaccine information should be part of the training curriculum in immunization area [1]. Many studies conducted in United Kingdom and United States of America revealed that, as with practicing physicians, parents and other medical staff possess varying knowledge regarding childhood vaccines [2,3].

There are various factors that are related to parental immunization knowledge and practices that are also associated with childhood immunization compliance. These factors include education of the parents, mother’s age at the time of delivery, mother’s race, number of preschool children, child order, and family income. In addition, immunization providers influence parental knowledge, practices, and decisions regarding immunization of children [4-9].

The level of parental education is the most important factor related to immunization knowledge and practices of parents. Most of the information regarding immunization risks and benefits is related to the level of parental education [8,9]. If parents receive good information about immunization, their worries and fears about vaccination will be eased. Previously published studies reported that mothers’ knowledge was found to be strongly associated with their educational level [5,8,9], while other studies found no correlation between immunization knowledge, attitudes, or practices (KAP) and the educational level of parents [7].

The effects of mothers’ age on immunization knowledge and practices were estimated. Mothers’ knowledge was significantly greater among the mothers who were older at the time of their child’s birth [5-8].

A previous study on immunization knowledge and practices indicated that race or ethnicity may contribute to inadequate knowledge, attitudes and practices of mothers [10].

Vaccination knowledge, attitudes and practices were correlated with family size and the number of siblings in each family. In a big household or in families with two or more children, it was found that the parents are less likely to have adequate knowledge or positive practices about the immunization of children [8].

The economic status of families were found to have a strong association with immunization knowledge, attitudes and practices of parents [4,8,9]. In addition, Zhang et al*.* found that health insurance and the frequency of watching TV or listening to the radio have a strong relationship with immunization knowledge, attitudes and practices of parents [9].

Immunization knowledge and practices of parents could be improved and developed in many ways that could increase the level of knowledge about the risks and benefits of vaccines. Health-care providers play an important role in child immunization. Familial knowledge and practices regarding vaccination mostly depend on vaccination providers for guidance on immunization timing and administration. In addition, immunization providers have positive effects on parental decisions related to vaccinations. Parents’ decisions regarding immunization can impact immunization rates, including access to vaccinations, the communication of risks and benefits, the maintenance of accurate vaccination records, and strategies for vaccination reminders [6,11-16].

## Methods

### Study design

A prospective cross-sectional study design was used to determine parental immunization knowledge and practices in Iraq, and to determine associations between parents’ knowledge – practice and familial data, and immunization provider’s characteristics, where the data were collected through a developed and validated interview-administered questionnaire [12]. This cross-sectional study was done among Iraqis’ parents that they attended to public health clinics and they interviewed by researchers for one time only, and the parents have children borne between 1st January 2003 and 31 June 2008 to ensure completeness of immunization histories.

### Questionnaire and data collection form

The questionnaire consisted of three parts: The first part of the questionnaire reflected on the demographics of respondents and family data. This part included father’s education, mother’s education, mother’s age at delivery, mother’s race, family marital status, number of preschool children, medical family history, family income, and the person who answered the questionnaire (father or mother).

An immunization provider characteristic which is the second part of the questionnaire included the number of immunization providers, provider types, and birth place. Many children completed their immunization within the same health institution, i.e. one provider, but some children received immunizations from many health institutions, considered as more than one provider.

The third and last part related to immunization knowledge and practice of Iraqi parents and the questionnaire was adapted from previous study [12]. Both the domains of knowledge and practices had 10 items each in their domains, single-choice questions from a multiple answers provided in each equation.

### Sampling: method and sample size

According to the Iraqi Ministry of Health Survey in 2008, a total of 116,000 children were to be immunized in Mosul [17]; the present study used this number as the total population from which the sample size was drawn. An automated software program (Raosoft sample size calculator for study: http://www.raosoft.com/samplesize.html) was used to calculate the sample size required for this study. With an accepted margin of error of 5% and a 95% confidence interval, the sample size required was 383. With the addition of 30% (as expected drop outs) to the estimated sample size in order to overcome erroneous results and increase the reliability of the results and the conclusion, the target sample size was 500 children.

Most of the Iraqi children received their immunization doses from general and public health clinics. Cluster sampling method was used in this study. Mosul divided to five parts or clusters depending on the Mosul map. One health clinics selected from each cluster in Mosul (five health clinics from five different area or clusters). These public health clinics operate three immunization days per week (Sunday, Tuesday, and Thursday), from 9.00 am. to 2.00 pm. Approximately 25 children attend these health clinics per day. Private clinics and general hospitals were not included in the setting for this study because the vaccines are providing by public clinics only and it is free for all.

### Study approval

The research proposal was submitted to the Iraqi Ministry of Health (MOH) in Baghdad, Iraq. Approval from the MOH (Reference no. 70667 in 15/12/2009) was obtained to facilitate the data collection by researchers from health clinics under Iraqi Ministry of Health before data collection was started. The parents and clinic staff were informed about the study aims and other details. The parents who were agreed to participate in the research had to sign the provided consent form before filling in the questionnaire.

### Data analysis

The data were analyzed using SPSS for windows (Statistical Package for Social Science) version 15.0 and the level of significance was set at less than 0.05 for all analyses. The Chi-square test was used to measure associations between nominal variables, and medians were calculated for continuous variables. Scoring of the questions was determined by giving one point (1) for each correct answer and zero (0) for wrong answers or no response (don’t know). The total knowledge scores and practice scores of the parents were calculated by adding up the scores for each question in the test. The total knowledge and practice scores ranged from 0 to 20, with higher scores indicating a higher level of immunization knowledge and practices. According to the median split method [18-20], parents with a total score of less than 12 (median) were considered as having inadequate knowledge and practices regarding child immunization and parents with scores from 12 to 20 were considered as having adequate knowledge and practices. This scoring method and categorization were used to identify the degree of parental immunization knowledge and practices in the current study.

## Results

A total of 528 parents were recruited in this study. The knowledge-practice scores ranged from zero to 20 and the result showed an average of 12.28 (SD = 2.95), with a median score of 12.

Using the categorization of the knowledge-practice scores explained in the median split method, we formed two groups of adequate and inadequate knowledge-practice of parents respectively.

Table 1 shows the frequency in percentage terms of the two groups. Out of the 528 parents who answered the questionnaire, 66.1% of the study population was found to have adequate knowledge-practice scores.

**Table 1:** Knowledge-practice scale (n = 528)

**Scale**	**Median**	**Frequency (%)**	
		**Inadequate**	**Adequate**
**KP **(20 questions)	12	179 (33.9)	349 (66.1)

Note: used median split method, inadequate when score < median, and adequate when score ≥ median.

Table 2 shows the 20 statements of knowledge and practice. The lowest correct answer (10.6%) was apparent in the question (8) related to the knowledge of vaccine storage. The highest correct answer (96%) was apparent in the statement related to the practice of vaccine recommendation (12).

**Table 2:** Correct and incorrect answer (n = 528)

**Statements**	**Frequency (%)**	
	**Correct answer**	**Incorrect answer**
1. Vaccination prevents disease.	447 (84.7)	81 (15.3)
2. Vaccination is for all ages.	187 (35.4)	341 (64.4)
3. There are different types of vaccines	374 (70.8)	154 (29.2)
4. Active immunization is a killed or weakened form of a disease-causing agent.	341 (64.6)	187 (35.4)
5. Passive immunization is an antibody from someone who was infected with the disease.	243 (46.0)	285 (54.0)
6. In some health situations, vaccines should not be given.	431 (81.6)	97 (18.4)
7. Vaccines need to be stored at more than 8 degrees Celsius and do not freeze.	206 (39.0)	322 (61.0)
8. The product should be used within 72 hours of the seal being broken.	56 (10.6)	472 (89.4)
9. There is a uniform immunization guideline for paediatric patients younger than two years.	343 (65.0)	185 (35.0)
10. Vaccination is harmful.	301 (57.0)	227 (43.0)
11. Are you in favour of vaccination?	496 (93.9)	32 (6.1)
12. Will recommend vaccination to others.	507 (96.0)	21 (4.0)
13. Vaccination should be initiated in the first week of age.	451 (85.4)	77 (14.6)
14. Were you informed about vaccination?	409 (77.5)	119 (22.5)
15. Did you read about vaccination in the media?	314 (59.5)	214 (40.5)
16. Did you see a television programme about vaccination?	367 (69.5)	161 (30.5)
17. Did you hear about vaccination on the radio?	214 (40.5)	314 (59.5)
18. Did you read about vaccination on the internet?	125 (23.7)	403 (76.3)
19. Did you obtain information about vaccination from an antenatal clinic?	348 (65.9)	180 (34.1)
20. Did you obtain information about vaccination from a maternity hospital or home?	212 (40.2)	316 (59.8)

Significant associations of knowledge-practice groups with father’s education level, mother’s education level, mother’s age at delivery, number of preschool children, questionnaire answered by parents, and family income were found (*p* < 0.05). Father’s and mother’s education level (>18 years) were found in a higher proportion within the parents with adequate knowledge-practice than other groups. Mother’s age at delivery of 20 to 29 years had a higher percentage (60.5%) of adequate KP than other groups. Families who had two or three preschool children were found in a higher proportion within the parents with adequate and inadequate knowledge-practice than other groups.

Families with income ≤400 US$ are more common within the group with inadequate KP than other groups, whereas families with income between 401 and 1000 US$ were more frequent (40.7%) in parents with adequate KP than other groups. Fathers were represented more often within adequate knowledge-practice, whereas mothers were represented more often within inadequate knowledge-practice. No significant association of knowledge-practice groups with mother’s race was found, as shown in Table 3.

**Table 3:** Association between KP groups and familial data (n = 528)

**Familial data**	**Knowledge-practice (%)**		**Total (%)**	**P**
	**Inadequate KP**	**Adequate KP**		
**Father’s education**				<0.001^a^
<13 yr	35 (19.6)	14 (4.0)	49 (9.2)	
13-18 yr	40 (22.3)	59 (16.9)	99 (18.8)	
>18 yr	104 (58.1)	276 (79.1)	380 (72)	
**Mother’s education**				<0.001^a^
<13 yr	55 (33.7)	25 (7.2)	80 (15.2)	
13-18 yr	38 (21.3)	87 (24.9)	125 (23.7)	
>18 yr	86 (48)	237 (67.9)	323 (61.1)	
**Mother’s age at delivery**				<0.001^a^
≤19	32 (17.9)	26 (6.5)	55 (10.4)	
20-29	104 (58.1)	211 (60.5)	315 (59.7)	
>29	43 (24)	115 (33)	158 (29.9)	
**Mother’s race**				0.701
Arabic	172 (96.1)	339 (97.1)	511 (96.8)	
Kurdish	7 (3.9)	10 (2.9)	17 (3.2)	
**Marital status**				0.036^a^
Married	173 (96.6)	347 (99.4)	520 (98.5)	
Single parent	6 (3.4)	2 (0.6)	8 (1.5)	
**Number of preschool children**				<0.001^a^
1	51 (28.5)	149 (42.7)	200 (37.9)	
2-3	125 (69.8)	179 (51.3)	304 (57.6)	
>3	3 (1.7)	21 (6.0)	24 (4.5)	
**Family income (US$)**				<0.001^a^
≤400	78 (43.6)	70 (20.1)	148 (28.0)	
401-1000	68 (38.0)	142 (40.7)	210 (39.8)	
1001-2000	29 (16.2)	100 (28.7)	129 (24.4)	
>2000	4 (2.2)	37 (10.5)	41 (7.8)	
**Questionnaire answered by**				<0.001^a^
Father	85 (47.5)	243 (69.6)	328 (62.1)	
Mother	94 (52.5)	106 (30.4)	200 (37.9)	
**Total**	**242 (100%)**	**286 (100%)**	**528 (100%)**	

Chi-square test, ^a^*p* < 0.05.

Significant associations of knowledge-practice groups with provider types were found (*p* < 0.05).

Children immunized in the public health system were the highest proportion among the parents with both adequate and inadequate KP. No significant associations of KP groups with numbers of immunization providers were found. Significant Associations of knowledge-practice groups with birth place groups were found (*p* < 0.05). Parents of child delivered in maternity hospital have higher proportion with adequate KP (47.5%) and inadequate KP (64.5%) than other groups, as shown in Table 4.

**Table 4:** Association between KP groups and immunization provider’s characteristics (n = 528)

**Provider’s characteristics**	**Knowledge-practice (%)**		**Total (%)**	**P**
	**Inadequate KP**	**Adequate KP**		
**Number of provider**				0.685
1	140 (78.2)	266 (76.2)	406 (76.9)	
>1	39 (21.8)	83 (23.8)	122 (23.1)	
**Providers types**				0.003^a^
Private clinic	0 (0)	10 (2.9)	10 (1.9)	
Public clinic	122 (68.2)	246 (70.5)	368 (69.7)	
Government hospital	24 (13.4)	22 (6.3)	46 (8.7)	
Private clinic & public clinic	9 (5.0)	37 (10.5)	46 (8.7)	
Private clinic & government hospital	12 (6.7)	17 (4.9)	29 (5.5)	
Public clinic & government hospital	12 (6.7)	17 (4.9)	29 (5.5)	
**Birth place**				<0.001^a^
General hospital	83 (46.4)	101 (28.9)	184 (34.8)	
Maternity hospital	85 (47.5)	225 (64.5)	310 (58.7)	
Maternity home	9 (5)	13 (3.7)	22 (4.2)	
Home	2 (1.1)	10 (2.9)	12 (2.3)	
**Total**	**242 (100%)**	**286 (100%)**	**528 (100%)**	

Chi-square test, ^a^*p* < 0.05.

## Discussion

The parental knowledge-practice (KP) scores were determined by summation of the knowledge score and practice score for each parent; about 66% of parents had adequate KP as they scored more than 12 out of the maximum score of 20 for the KP test. This could be because of an increase in sources of vaccination and health information represented by television, the internet and other sources. Before 2003, many restrictions in Iraq were imposed on the media, especially on television and internet, whereas an increase in the number of international medical and scientific TV channels as well as an increase in internet users after 2003 could probably contribute to the increase in parents’ immunization practice and in immunization knowledge.

The results of this study was similar to other findings in an Italian study [5] in which more than half of respondents had adequate KAP, and is supported by a study in India [16] that found parental knowledge regarding vaccination adequate.

On the other hand, most of the parents in different countries had inadequate or limited immunization knowledge or bad immunization practice. In India [21,22] Indian mothers had limited information about vaccine-preventable diseases. Approximately 60% of Chinese mothers did not have good knowledge regarding immunization for children [7,8]. Studies in Canada [23] and Germany [24] showed most parents complained about insufficient information about childhood immunization. The low scores of knowledge and practice in many countries might be due to variety of factors could affecting parental information and knowledge, but the important factors are related to the type of immunization provider, sources of information, family income, immunization cost and other barriers.

A significant association was noted between immunization KP and education level of fathers and mothers. The association between knowledge level and educational level was consistent with previous studies regarding the evaluation of vaccination knowledge [5,6,8,9]. This could be because fathers or mothers with higher educational level occupied upper socioeconomic stratum and find it easy to obtain knowledge from the press, books and internet.

Interestingly, many parents graduated from medical college and science college showed adequate knowledge on immunization. In contrast, an Indian study [16] and Spanish study [25] found that parents with high education levels had inadequate knowledge regarding vaccination.

Parents’ KP scores were associated with mother’s age. This finding is supported by other studies in which younger mothers had higher KP scores [7,8], There are many reasons; older parents might have had better education, attended talks and used the internet as a source of information, reflected in their higher knowledge rate, and the immunization provider may be more likely to teach older parents, considering that older parents have good cognitive capacity.

An insignificant association of immunization KP with mother’s race was found. The findings are similar to findings in another study in China [8] in which insignificant association was found between immunization knowledge and mother’s race. This result is normal and it reflect the social environment in Iraq represented by no differentiated between Arab and Kurd peoples in education, health, childcare and other area of daily life.

A Canadian researcher found a significant relationship between marital status and immunization compliance in Canadian children [26]. This study showed a significant association between KP of immunization and marital status. This result is not surprising because one of the parents might provide the information to another parent and increase the source of information. In addition, the married parents had a higher socioeconamic status than divorced or widowed parents.

Studies in different countries [27-30] found that association was significant and there was a negative correlation between child number or sibling number in each family or family size and child’s immunization rate. A significant association of parent’s KP with number of children was found in this study. When the number of children increases in a family, the time needed for health care for each child will decrease and the time needed to receive immunization information from health clinics will also decrease. In addition, the family socioeconomic status will increase when the family’s size decreases. The results are inconsistent with other studies [8,15] in which the KP scores increased when the family size increased because the experience of children’s immunization improved when the family’s size increased.

Low family incomes as well as limited parental education are problems faced by many parents and can adversely affect their immunization knowledge and practice, and their ability to complete their children’s vaccination. Lower family income could be a barrier to effective communication between immunization providers and parents. These results, similarly to other studies in developing countries, show that mothers’ knowledge, attitude and practice are positively correlated with families’ monthly income [9,31].

Significant association between parent’s gender and immunization KP was found in the current study. This study was inconsistent with that of Wang et al. (2007) who found that gender did not have significant association with immunization KAP, but that males had higher KAP scores than females. From these findings, this study suggests the association and effect of gender might relate to paternal education. More than 60% of the questionnaires were answered by fathers whereas less than 40% of questionnaires were answered by mothers.

Immunization providers have a role to play in the decision of parents to immunize their children by providing complete information on vaccines, especially their risks and benefits. For this reason, the immunization providers have an important role in increasing knowledge about the importance of vaccination. Many studies have shown that the knowledge level amongst parent and medical staff regarding vaccination is variable [32-36].

Insignificant associations of immunization KP with parents who had children immunized by one or more immunization providers was determined. The results relating to the provider number does not affect immunization knowledge and practice received by parents from immunization providers because immunization information will be the same from one provider or two providers or more in the same type of health institution.

There are three types of immunization providers in Iraq; public health clinics, private health clinics and general or government hospitals. Most children are vaccinated in public health clinics because they are free and available in many places. A significant association between parent’s KP and immunization providers was shown. The parents can receive immunization recommendations and develop their immunization experience when their children are vaccinated in private clinics more than by other types of immunization providers because the private immunization provider can spend more communication time with parents, the number of children visiting this type of clinic is very low because of the high cost, and the parents receive enough information regarding risk/benefits that is of a high quality Birthplace is one of the important factors affecting vaccination rate, knowledge and practice because most children are vaccinated with their first dose (BCG) in their place of birth. Also, the parents will receive immunization recommendations and information during the first days in the place of birth because the mother and her child stay in the hospital for two or three days after delivery and this provides an opportunity for good communication with medical staff.

A significant association of immunization KP with children’s birth place was found. These results suggest parents receive good and adequate knowledge and practice regarding immunization from maternity hospitals more than from other places; the main reason for this finding is that medical staff (physicians, pharmacists, nurses and others) in maternity hospitals know more about child health and women’s health care than staff in other hospitals. For this reason, parents receive specific and concentrated knowledge from maternity hospital staff.

## Conclusion

The parents’ KP in this study was associated with many factors included family and immunization providers. Health care providers’ factors should be considered when an educational intervention is planned because open and effective provider communication can facilitate better immunization compliance, knowledge and practice. Improving communication between parents and immunization provider will engage the parents in decision-making clarifying the importance of immunization and highlight the value of immunization compliance. There is a need to increase awareness and knowledge about the benefits and importance of vaccination, as well as the harmful consequences of non-complete immunization. A planned educational programme is needed; the educational level of the parents needs to be taken into consideration when the programme is planned, especially as regards those with a lower educational level.

### Study limitation

This study only targeted children younger than two in five clinics in Mosul, who may not represent all Iraqi children. The convenient sampling and cohort design further limit the generalization of the findings to the entire Iraqi population.

## Competing interests

The authors declare that they have no competing interests.

## Authors’ contributions

All authors have made substantial contributions to the conception of the study, drafting the article, and final approval of the version to be submitted. OQ, MB, and MG conceived and designed the study. OQ and MR did the electronic search for the relevant articles and drafted the manuscript. MR and RM analyzed the data. OQ and SJ revised and edited the manuscript. RM and SJ prepared the manuscript for publication. All authors have read and approved the final submitted manuscript.

## Pre-publication history

The pre-publication history for this paper can be accessed here:

http://www.biomedcentral.com/1471-2431/14/29/prepub
